# Prognostic Value of CXCL13, CCL11, and CCL20 Chemokines in Multiple Sclerosis

**DOI:** 10.3390/biomedicines13010040

**Published:** 2024-12-27

**Authors:** Işıl Peker, Hacer Eroğlu İçli, Belgin Mutluay, Burcu Yüksel, Zeynep Özdemir, Mesrure Köseoğlu, Aysu Şen, Dilek Ataklı, Aysun Soysal, Musa Öztürk

**Affiliations:** 1 Department of Neurology, Edirne Sultan 1. Murat State Hospital, 22030 Edirne, Turkey; 2 Department of Biochemistry, Bakirkoy Research and Training Hospital for Psychiatry, Neurology and Neurosurgery, 34147 Istanbul, Turkey; 3 Department of Neurology, Bakirkoy Research and Training Hospital for Psychiatry, Neurology and Neurosurgery, 34147 Istanbul, Turkey; 4 Department of Neurology, Kanuni Sultan Suleiman Research and Training Hospital, 34303 Istanbul, Turkey

**Keywords:** relapsing–remitting multiple sclerosis, B lymphocyte chemoattractant molecule, eotaxin-1, macrophage inflammatory protein 3-alfa, biomarkers

## Abstract

**Objective:** The course of relapsing–remitting multiple sclerosis (RRMS) is highly variable and there is a lack of effective prognostic biomarkers. This study aimed to assess the potential prognostic value of the chemokines B lymphocyte chemoattractant molecule (CXCL13), eotaxin-1 (CCL11), and macrophage inflammatory protein 3-alpha (CCL20) in RRMS. **Methods:** Forty-two patients with MS were enrolled, along with 22 controls, 12 of the controls were idiopathic intracranial hypertension (IIH) patients, and 10 of the controls were other neurologic diseases (OND). Chemokine levels were measured using enzyme-linked immunosorbent assay (ELISA) in serum and cerebrospinal fluid (CSF) samples. **Results:** No significant differences were observed among the groups in serum levels of CXCL13, CCL11, and CCL20 (*p* = 0.509, *p* = 0.979, *p* = 0.169, respectively). CSF CXCL13 levels were significantly higher in the OND group (*p* = 0.016). A PATH analysis showed CSF CXCL13 was significantly associated with new T2 hyperintense lesions on brain magnetic resonance imaging (*p* < 0.001), and baseline serum CCL11 levels were associated with EDSS (*p* = 0.030), implying its potential role in indicating neurodegenerative processes and possible progression risk. Serum CCL20 correlated with EDSS (*p* = 0.002) and lesion burden (*p* < 0.001), reflecting disease severity. **Conclusions:** These findings suggest that CSF CXCL13 could serve as a useful biomarker for predicting active disease in RRMS, while follow-up serum CCL11 may assist in identifying progression. Although these chemokines are not specific to MS, higher levels may signal disease activity, severity, and transition to more progressive stages.

## 1. Introduction

Multiple sclerosis (MS) is a chronic disease characterized by demyelination and axonal degeneration in neurons of the central nervous system (CNS), often manifesting in recurring attacks [1]. Given its potential to cause disability in young adults, initiating effective treatment is crucial. However, due to the heterogeneity of the disease, predicting progression and selecting the most appropriate treatment can be challenging. The risk of serious side effects and heterogeneous responses to disease-modifying therapies also necessitate a case-based approach in treatment selection [2]. Therefore, biomarkers that can predict neuroinflammation and progression are required.

One promising avenue is the use of chemokines, which play an undeniable role in MS pathogenesis, as potential serum- and cerebrospinal fluid (CSF)-based biomarkers for monitoring disease activity and therapy response [3]. Chemokines are a family of proteins responsible for leucocyte recruitment to sites of inflammation. In MS, they facilitate the migration of immune cells across the blood–brain barrier (BBB) and amplify the inflammatory response in the CNS [4]. CXCR3 and CCR5 (receptors for CCL11) are expressed by T cells in MS lesions, while their ligands are expressed by microglia and vascular endothelial cells, reflecting T helper1 (Th1) cell infiltration [5]. Several studies have shown that active demyelinating plaques, as well as serum and CSF of MS patients, express higher levels of CXCR3 and CCR5, while CCR4 expression, associated with T helper 2 (Th2) cells, is significantly reduced. This suggests that Th1 cells dominate MS pathogenesis, with a shift toward Th1 activity during disease flare-ups [5,6,7].

Several mechanisms contribute to MS development, including the migration of autoreactive Th1 cells across the BBB and Th17 cells expressing high levels of CCR2 (a receptor for CCL11), CCR4, and CCR6. To facilitate the early entry of Th17 cells across the BBB, CCL20, a ligand for CCR6, is produced by choroid plexus epithelial cells. As a consequence, disease onset may occur. Studies using experimental autoimmune encephalomyelitis (EAE) models have shown that the T cell infiltration into the CNS is reduced in CCR6-null mice, suggesting that Th17 cells lacking CCR6 cannot effectively invade the CNS [8,9].

The first chemokine investigated, CXCL13, plays a crucial role in B lymphocyte migration through its binding to CXCR5. Under pathologic conditions, CXCL13 is believed to trigger inflammation, leading to the production of macrophages, dendritic cells, and microglia. Several studies have found that CXCL13 levels in both serum and CSF are elevated in active MS cases, indicating its potential as a marker of inflammation [10,11,12].

CCL11, originally identified as a potent eosinophil chemoattractant, plays a significant role in conditions such as asthma, inflammatory bowel disease, and atopic dermatitis [13]. After binding to its receptor, CCL11 not only directs eosinophils to inflammation sites but also affects the functions of basophils, Th2 lymphocytes, and mast cells [14]. Studies have shown that serum CC11 levels are lower in relapsing–remitting multiple sclerosis (RRMS) patients compared to healthy controls but tend to increase in secondary progressive MS (SPMS), suggesting a shift from Th1 to Th2-dominant immune responses as the disease progresses. These findings suggest the potential of CCL11 as a biomarker for distinguishing between inflammatory and progressive phases of MS [15,16].

CCL20 is a chemotactic molecule that primarily targets lymphocytes and, to a lesser extent, neutrophils. It is produced by endothelial cells and macrophages in response to stimuli such as IL-6, IFN-γ, TNF-α, and IL-1ß. Its receptor, CCR6, is specific to CCL20. The binding of CCL20-CCR6 mediates the chemotaxis of immature dendritic cells, effector/memory T lymphocytes, and B lymphocytes [17]. CCR6-CCL20 binding enables Th17 lymphocytes to cross the BBB, playing a significant role in the onset of MS. Th17 cells secrete IL-17, which enhances pro-inflammatory responses and increases CCL20 transcription, further promoting T lymphocyte migration into the CNS and exacerbating disease activity [8,18]. Additionally, CCL20 binding to receptors on Th17 cells and its role in inducing CXCL13 production in B cells contribute to an inflammatory cascade that amplifies MS pathology [19].

This study aimed to assess whether these chemokines (CXCL13, CCL11, and CCL20) could serve as prognostic biomarkers in MS.

## 2. Materials and Methods

Serum and CSF samples were collected from 64 subjects divided into three groups: MS (*n* = 42), idiopathic intracranial hypertension (IIH, *n* = 12), and other neurological disease (OND, *n* = 10). MS cases met the McDonald’s criteria [20], and the control groups were age and sex-matched to MS cases. The OND group included patients with headache (*n* = 4), psychosis (*n* = 3), cerebrovascular disease (*n* = 2), and axonal polyneuropathy (*n* = 1).

Clinical and demographic data collected included age, age of onset, disease duration (time from first clinical symptoms to lumbar puncture), Expanded Disability Status Scale (EDSS) scores, number of clinical relapses, and CSF findings (CSF cell count, protein levels, oligoclonal bands, IgG index, and Qalbumin). White matter lesion counts on the initial T2-weighted brain magnetic resonance imaging (MRI) and new lesions in one-year follow-up MRI were also recorded as neuroimaging data.

The cases were divided into high CSF white cell count (≥10/mm^3^) and normal CSF cell count (<10/mm^3^). Oligoclonal IgG bands were assessed by isoelectric focusing and evaluated as described [21]. The CSF IgG index was calculated using the formula: IgG index = CSF IgG: serum IgG/CSFalbumin: serum albumin. The albumin quotient also was calculated as QAlbumin = CSF albumin (mg/L): serum albumin (g/L).

At the time of the first sampling, none of the MS cases had received intravenous steroid or disease-modifying treatments. After one year of follow-up, additional serum samples were collected from the MS group. During this period, the patients were treated with dimethyl fumarate (*n* = 10), teriflunomide (*n* = 5), fingolimod (*n* = 4), natalizumab (*n* = 2), cladribine (*n* = 2), pegylated- interferon (*n* = 1), glatiramer acetate (*n* = 1), ocrelizumab (*n* = 1), and azathioprine (*n* = 1). Four cases did not receive any treatment.

Serum and CSF samples were centrifuged at 3000 rpm for 10 min, and then samples were separated and transported into two Eppendorf tubes to be stored at −80 °C after sampling without delay. Following the manufacturer’s instructions, serum and CSF chemokine levels were measured using commercially available enzyme-linked immunosorbent assay (ELISA) kits (Human BLCA, human ECF/CCL11, and human MIP-3α, Elabscience, Wuhan, China). Each measurement was performed in duplicate.

The study was approved by the Istanbul Bakirkoy Dr. Sadi Konuk Education and Research Hospital Clinical Research Ethical Board (protocol number 2021/251) and received permission from the Scientific Research Platform of the Health Department.

Statistical analyses were performed using IBM SPSS V23 and IBM SPSS AMOS V24 (SPSS Inc., Chicago, IL, USA). The Kolmogorov–Smirnov test and the Shapiro–Wilk test were used to assess the normality of variables. For normally distributed data, one-way Analysis of Variance (ANOVA) was used for comparisons across three or more groups. For non-normally distributed data, the Kruskal–Wallis H test was utilized to compare median values in three or more groups. Multiple comparisons were analyzed using the Dunn test. For the comparison of non-normally distributed parameters measured at two time points, the Wilcoxon test was used. Spearman’s rho correlation coefficient was performed to examine the relationship between non-normally distributed parameters. PATH analysis was used to investigate the relationship between independent variables and dependent variables. Data were expressed as mean ± SD, median (minimum–maximum). A *p*-value of less than 0.05 was considered statistically significant.

## 3. Results

### 3.1. Demographics and Clinical Characteristics of the Groups

The mean ages of MS, IIH, and OND groups were 31.6 ± 8.7 years, 35.1 ± 11.9 years, and 36.3 ± 9.3 years, respectively (*p* = 0.254). In the MS group, 29 (66%) patients were female, while 11 (92%) in the IIH group and seven (70%) in the OND group were female (*p* = 0.249). There were no significant differences in age or sex between the groups (Table 1).

Routine CSF cell counts were within normal ranges for all groups. The MS group had higher median CSF protein levels and IgG index values compared to the IIH group, although no significant differences were observed between the MS and OND groups (Table 2). The oligoclonal band (OCB) was positive in 35 (85%) of the MS cases. In the IIH group, seven cases were analyzed for OCB, and all were negative, as was the one case analyzed in the OND group. In the MS group, 30 (71%) cases had a disease duration of less than two years, and the median EDSS score was 1 (Table 1). Only 11 (26%) cases had an EDSS score higher than 1. After one year of follow-up, 11 patients discontinued, leaving 32 monitored for one year. Among these, only one case showed an increase in EDSS, and five follow-up MRIs revealed new T2 lesions.

### 3.2. Serum and CSF Chemokine Levels

**Figure 1:**
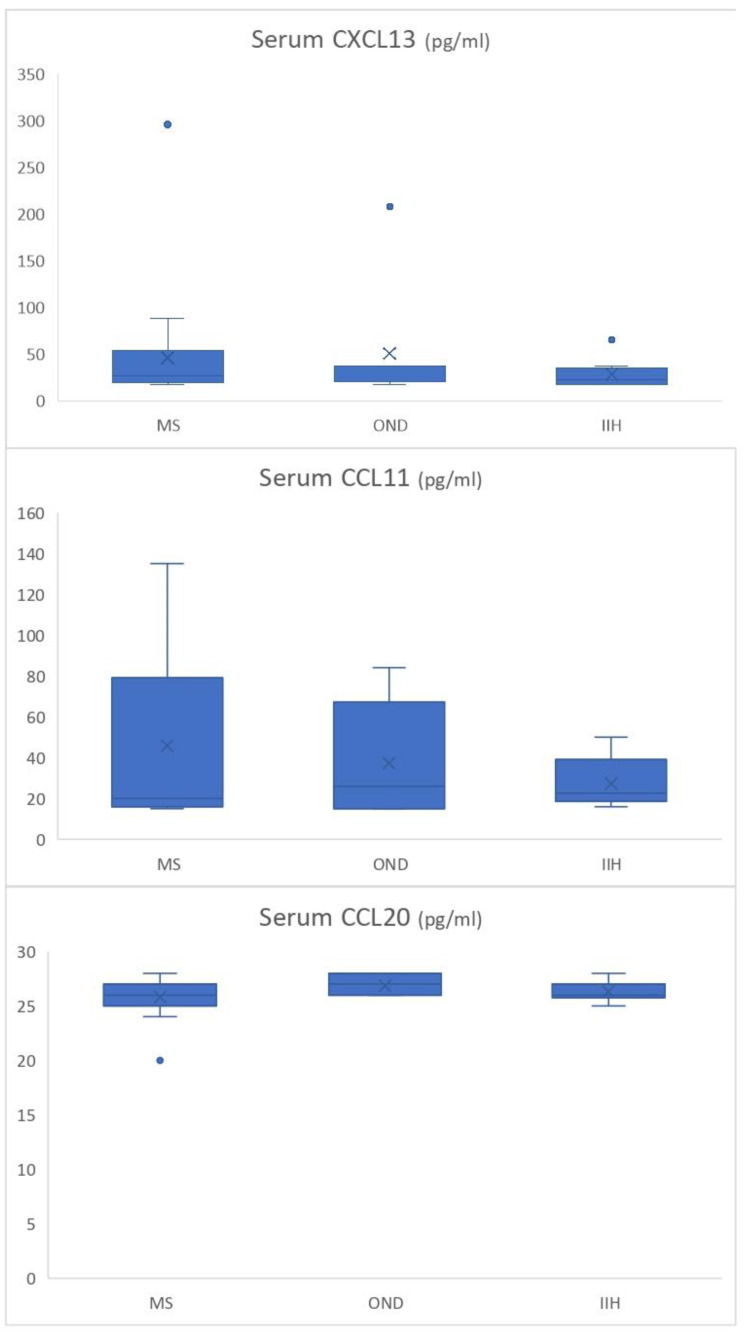
The box plots illustrate baseline serum CXCL13, CCL11, and CCL20 levels (pg/mL = picograms/milliliter) in MS (*n* = 23), IIH (*n* = 10), and OND (*n* = 7) groups.

**Figure 2:**
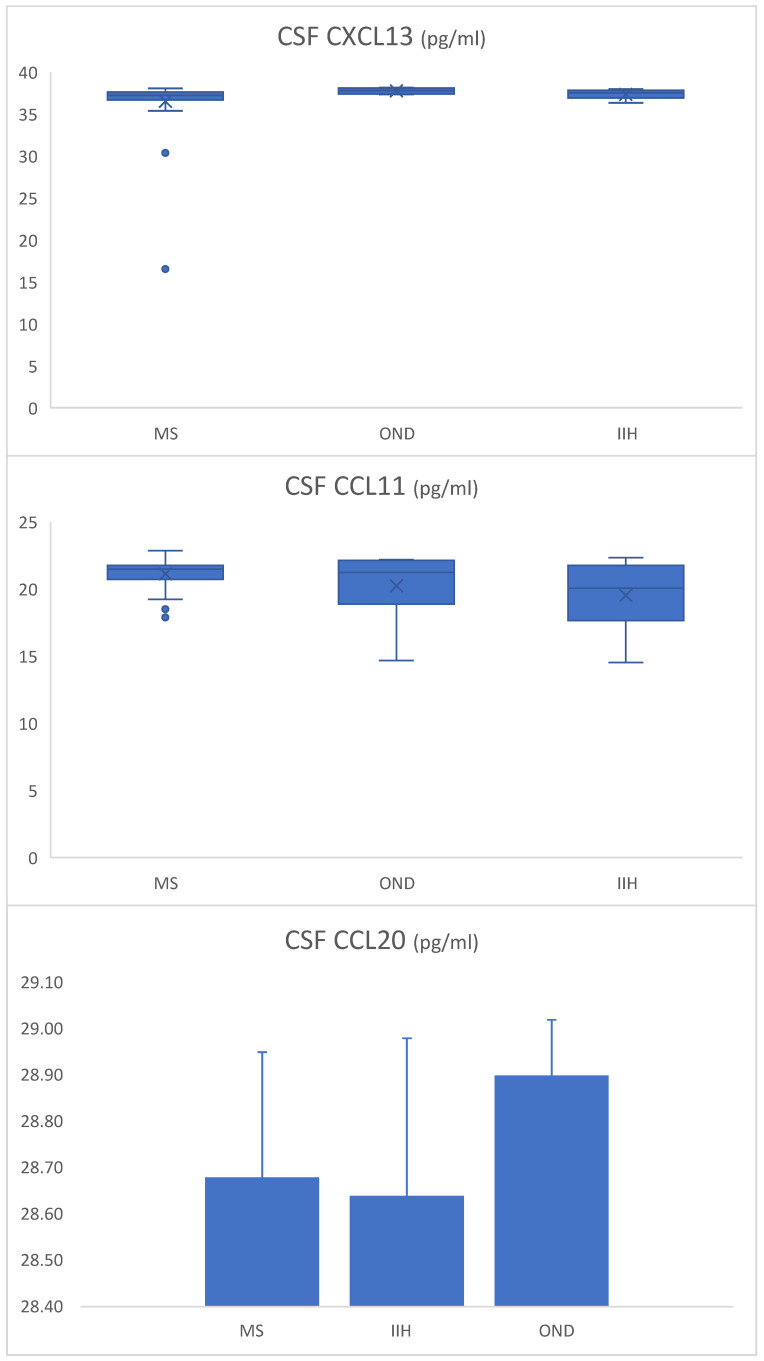
CSF CXCL13, CCLL11, and CCL20 levels (pg/mL = picograms/milliliter) in MS (*n* = 42), IIH (*n* = 12), and OND (*n* = 10) groups.

No statistically significant differences were found in median serum chemokine levels (Figure 1 and Table 3). A statistically significant difference in median CSF CXCL13 levels was observed between the groups (*p* = 0.016, Figure 2 and Table 3). Pairwise comparisons using the Bonferroni test showed that, although the median chemokine level in the OND group was numerically similar to the other groups, it was significantly higher than that of both the MS and IIH groups. No significant differences were found between groups in CSF CCL11 and CSF CCL20 levels (*p* = 0.173 and *p* = 0.085, respectively).

Serum chemokine levels were measured in 19 of the 32 follow-up cases. As shown in Table 4, there were no statistically significant changes between baseline and follow-up levels, nor were there significant differences based on the immunomodulatory treatments received. Although CCL11 levels increased slightly at follow-up, this change was not statistically significant, and other chemokine levels remained stable.

### 3.3. Correlations Between Serum and CSF Chemokine Levels

**Figure 3:**
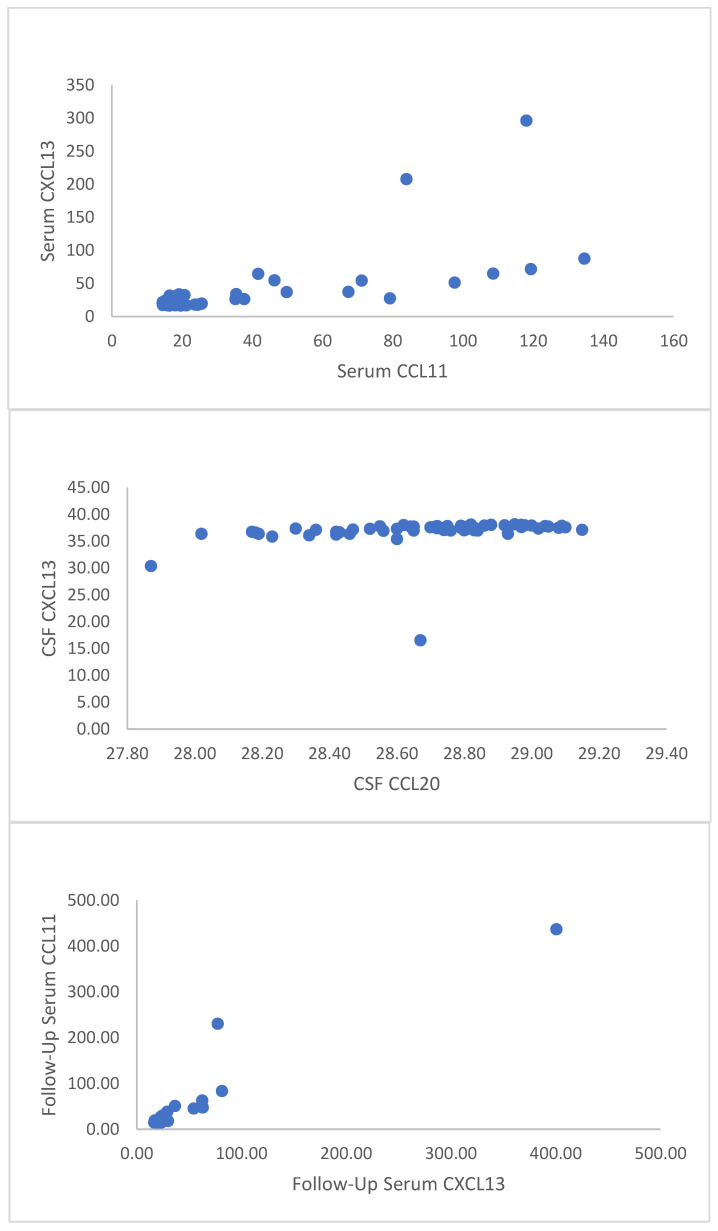
Correlation graphics between baseline serum CXCL13 and serum CCL11 (**top**), CSF CXCL13 and CSF CCL20 (**middle**), follow-up serum CXCL13 and follow-up serum CC11 (**bottom**).

Correlation analysis revealed a statistically significant strong correlation between baseline serum CXCL13 and serum CCL11 levels (r = 0.669, *p* < 0.001) and between follow-up serum CXCL13 and serum CCL11 levels (r = 0.835, *p* < 0.001). A statistically significant moderate correlation was observed between CSF CXCL13 and CSF CCL20 levels (r = 0.585, *p* < 0.001) (Figure 3). No significant relationships were observed among the other parameters (Table 5).

### 3.4. Correlation Between Serum/CSF Chemokine Levels and Clinical Findings

A structural equation model was performed illustrating the relationships between various clinical and laboratory parameters—age at onset, disease duration, CSF protein, EDSS, presence of OCB, IgG index, Qalb, occurrence of relapse, number of T2-weighted white matter (WM) lesions on the initial MRI, and the presence of new T2 WM lesions on follow-up MRI—and CSF CXCL13, CCL11, and CCL20 levels in the MS group.

**Figure 4:**
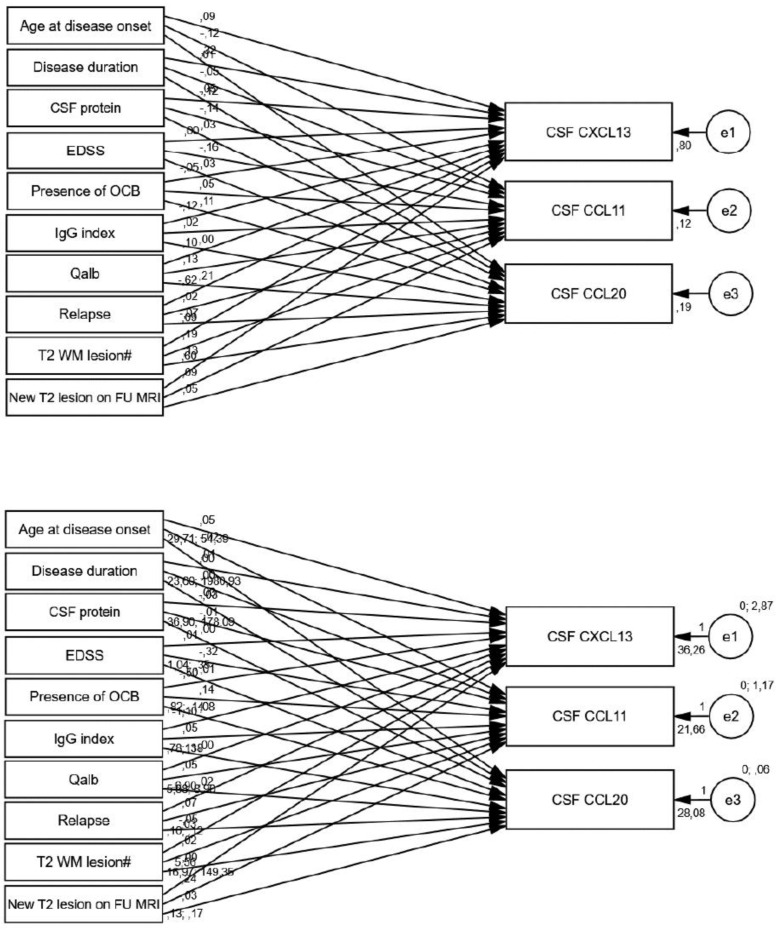
Standardized (**top**) and unstandardized (**bottom**) path coefficients of PATH analysis of CSF chemokines.

The presence of relapse was significantly associated with lower CXCL13 levels (*p* < 0.001) (Table 6, Figure 4), decreasing by 6.902 pg/mL (picogram/milliliter). In contrast, the presence of new T2 WM lesions on follow-up MRI was significantly related to higher CXCL13 levels (*p* < 0.001), increasing by 5.558 pg/mL.

**Figure 5:**
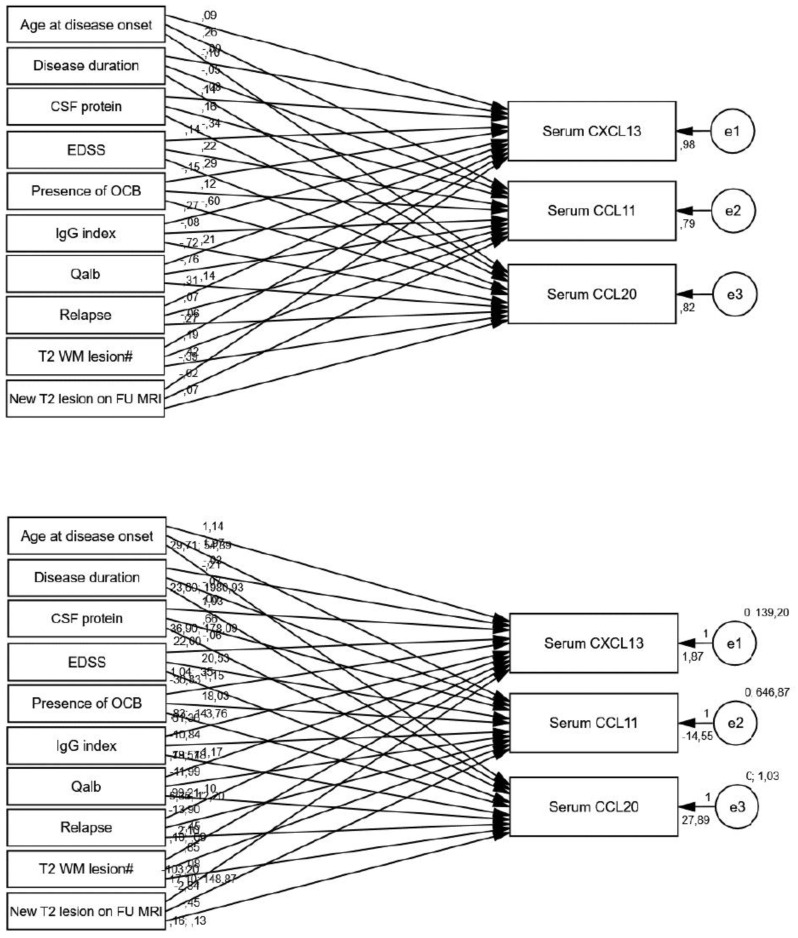
Standardized (**top**) and unstandardized (**bottom**) path coefficients of PATH analysis of baseline serum chemokines.

All of the factors showed statistically significant relationships with baseline serum CXCL13 levels (Table 7, Figure 5). Each additional year of the onset age of MS corresponded to a 1.135 pg/mL increase in CXCL13 (*p* = 0.012), and each one-unit increase in EDSS was associated with a 22.003 pg/mL increase in CXCL13 (*p* < 0.001). In addition, patients who experienced a relapse had significantly higher CXCL13 levels compared to those without relapse (*p* < 0.001), demonstrating that cases with relapse had CXCL13 levels 99.212 pg/mL higher than those without relapse. A one-unit increase in the T2 WM lesion count on the initial MRI was associated with a 2.097 pg/mL increase in CXCL13 (*p* < 0.001).

Conversely, longer disease duration (ß = −0.210, *p* = 0.005), higher Qalb values (ß = −19.565, *p* < 0.001), and the presence of oligoclonal bands (ß = −36.826, *p* < 0.001) were associated with decreases in CXCL13. Each additional month of disease duration is linked to a 0.210 pg/mL decrease in CXCL13. The path coefficient examining the presence of new T2 WM lesions on follow-up MRI was statistically significant (*p* < 0.001). Patients with new T2 WM lesions exhibited CXCL13 values that were 103.203 pg/mL lower than those without these lesions.

Similar to the observed patterns in serum CXCL13 levels, serum CCL11 levels also demonstrated a tendency to increase with both age at disease onset (*p* = 0.010) and EDSS (*p* = 0.030), while higher Qalb values (*p* < 0.001) were associated with lower CCL11 levels. Each one-unit increase in EDSS resulted in a 20.532 pg/mL increase in CCL11.

For CCL20, higher EDSS (*p* = 0.002), IgG index (*p* = 0.023), and the T2 WM lesion count on the initial MRI (*p* < 0.001) were each significantly associated with increased CCL20 levels. In contrast, higher CSF protein levels (*p* < 0.001) and the presence of oligoclonal bands (*p* < 0.001) were associated with reduced CCL20 values.

**Figure 6:**
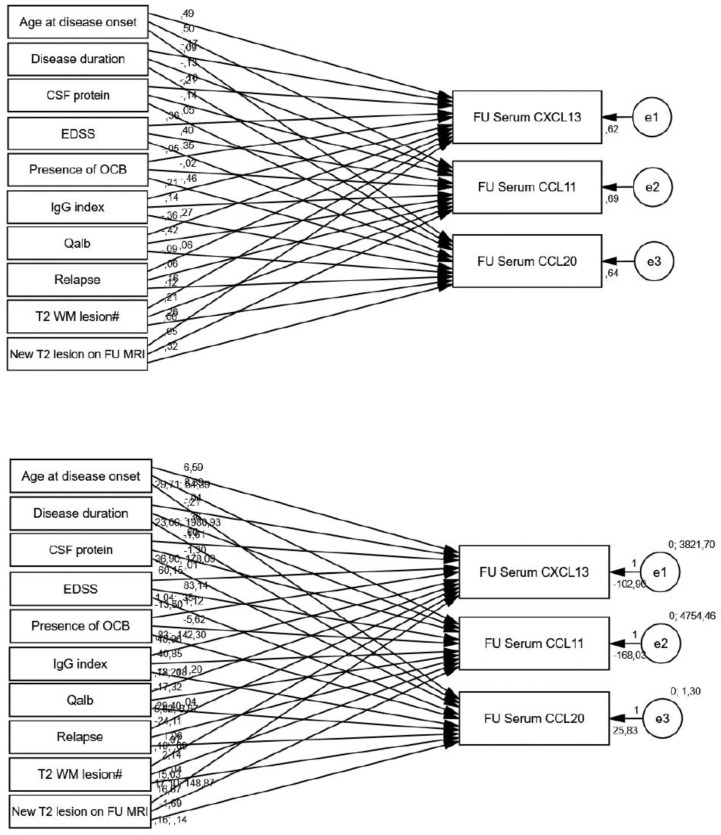
Standardized (**top**) and unstandardized (**bottom**) path coefficients of PATH analysis of follow-up serum chemokines.

Figure 6 presents the results of the structural equation model based on follow-up serum chemokine levels. Increasing age at disease onset was significantly associated with higher follow-up CXCL13 (*p* < 0.001) and CCL11 levels (*p* < 0.001). In contrast, elevated Qalb values were significantly associated with reductions in follow-up CXCL13 (*p* = 0.016) and CCL11 (*p* = 0.002). As shown in Table 8, higher EDSS were also significantly related to elevated CXCL13 (*p* = 0.010), CCL11 (*p* = 0.002), and CCL20 (*p* = 0.007). The T2 WM lesion count on the initial MRI also correlated with slightly higher CCL20 levels (*p* = 0.044). In contrast, patients exhibiting new T2 WM lesions on follow-up MRI had CCL20 levels that were 1.686 pg/mL lower than those without new lesions (*p* = 0.012).

## 4. Discussion

This study examined serum and CSF chemokine levels in MS cases that mostly had a disease duration of less than two years and compared them with two control groups: IIH patients and OND cases. We divided the control group into two based on recent theories emphasizing the role of dysregulated immune function in IIH pathogenesis, which has been linked to a pro-inflammatory cytokine profile [22]. We specifically investigated whether there was a difference between MS and IIH cases. However, our findings revealed no significant differences in serum chemokine levels between the groups.

Serum CXCL13 was found to be increased with several systemic active diseases such as rheumatoid arthritis, systemic lupus erythematosus, and infections [23,24]. Higher levels of this serum marker were also associated with active MS [11,25]. Our model showed that baseline serum CXCL13 was found to be increased with EDSS, clinic relapse, and T2 WM lesion count but decreased with prolonged disease duration and new T2 lesions on follow-up MRI. On the contrary, CSF CXCL13 was found to be increased with new T2 lesions on follow-up MRI. In line with findings related to baseline chemokines, follow-up serum CXCL13 was also found to be increased with EDSS. This disparity between serum and CSF CXCL13 could be explained by the possibility of the compartmentalized nature of the inflammatory response in CNS. In the literature, there is supporting evidence that CSF CXCL13 is most likely derived from the CNS, not crossed from blood, while elevated serum CXCL13 with little or no change in CSF levels may reflect inflammation localized outside the CNS [26]. CSF levels are primarily influenced by the local CNS microenvironment, while serum levels represent systemic immune activity. Serum CXCL13 increased during relapse, and its levels subsequently decreased when new lesions formed on follow-up MRI, suggesting a transient immune surge that is not associated with persistent chemokine production at the CNS level. On the other hand, the opposite pattern observed in the CSF suggests that local inflammatory processes within the CNS evolve differently, possibly as a result of ongoing lesion formation and localized immune cell infiltration.

Previous studies have proposed that CSF CXCL13 serves as a biomarker for inflammation and MS disease severity [10,27]. Elevated levels of CSF CXCL13 have been linked to higher relapse rates, EDSS scores, and the number of lesions detected by MRI [28]. We found a positive correlation between new T2 hyperintense lesions and CSF CXCL13 levels. CXCL13 has been identified in active demyelinating lesions and perivascular infiltrates, suggesting its role in attracting B and T cells into inflamed MS lesions [29]. Additionally, our findings showed a strong correlation between CSF CXCL13 and CCL20, supporting the theory that CCL20–CCR6 interactions promote CXCL13 production, which subsequently recruits B lymphocytes during early MS pathogenesis [19]. Furthermore, this suggests that monitoring CCL20 with CXCL13 may help to provide stronger prognostic information about the disease course.

Although the mean CSF CXCL13 levels were similar across all groups, the OND group displayed statistically higher levels. The composition of the control cases may explain this discrepancy. A study by Swiderek-Matysiak et al. found no significant differences in CSF CXCL13 between MS patients and a control group comprising systemic inflammatory diseases with CNS involvement, cerebrovascular diseases, and other non-inflammatory neurological diseases [30]. High CSF CXCL13 levels have also been observed in other neurological conditions, including autoimmune encephalitis, paraneoplastic syndromes, active neuroborreliosis, and neurosyphilis. In most cases, the level of this marker was positively correlated with disease activity [31,32,33,34]. Therefore, we can conclude that while its intrathecal production is not specific to MS, it is a sensitive marker of acute CNS inflammation.

Regarding CCL11, studies have shown increased expression in choroid epithelial plexus cells in aging mice [35]. In vitro experiments conducted with neural cells have demonstrated that CCL11 activates the production of reactive oxygen species in microglia, triggering excitotoxic neural cell death. The data suggest that CCL11 may inhibit endogenous repair mechanisms or exacerbate the ongoing neurodegeneration process [36,37]. It has also been shown that patients with neurodegenerative diseases like Alzheimer’s, Huntington’s, and amyotrophic lateral sclerosis have significantly higher concentrations of CCL11 in their serum or CSF. Therefore, CCL11 levels may be a useful biomarker for predicting the progression of these diseases [37].

CCL11 was shown to be higher in progressive MS cases, with levels correlated with EDSS scores and disease duration [16,37]. Therefore, CCL11 could be a candidate biomarker for monitoring disease progression [38]. We observed a positive correlation between EDSS and both baseline and follow-up serum CCL11, although no significant differences were found in serum and CSF levels in our cohort, likely due to the lack of progressive MS cases and healthy controls. In another study, a control group with ONDs also revealed no significant differences, leading to the conclusion that the chemokines are not specific to MS [39]. Nevertheless, each one-unit increase in EDSS resulted in an 83.137 pg/mL increase in one-year follow-up serum CCL11, which is noteworthy, as increased CCL11 levels of more than approximately 80 pg/mL at follow-up could indicate a risk of disease progression.

Serum CCL20 has been correlated with MS severity [38], reflecting inflammation by promoting pathogenic T cell migration to the CNS. In our study, we also observed each one-unit increase in EDSS resulted in an increase in both baseline and follow-up serum CCL20.

Similar to the baseline CXCL13 levels, follow-up serum CCL20 was also found to be increased with EDSS and T2 WM lesion count but decreased with new T2 lesions on follow-up MRI. In studies conducted with serum samples from MS patients, CCL20 levels were found to be elevated compared to healthy controls, while no significant differences were observed in CSF samples [40,41]. Similarly, Burman and colleagues investigated specific CSF cytokine profiles for MS, finding no significant differences between CSF CCL20 levels in MS and OND cases [42]. Consistent with these results, we did not observe any significant differences. Thus, it can be concluded that serum CCL20 may not be specific to MS but a sensitive biomarker that reflects the clinical severity.

We identified a statistically significant positive correlation between age and the chemokines. The literature suggests that aging is associated with aberrant gene regulation in cells, leading to a pro-inflammatory state that can affect systemic cytokine and chemokine levels [43]. However, due to the lack of data, we cannot draw definitive conclusions regarding the temporal variations of chemokines. Given that our study did not include a healthy control group, it would be inappropriate to make broad interpretations based on our data. Nonetheless, a study examining blood samples from donors reported that CCL11 levels increased with age, independent of the duration of the samples [44].

Both baseline and follow-up serum CXCL13 and CCL11 were positively correlated, and both tended to decrease as Qalb values increased. Qalb generally reflects the blood–brain barrier integrity and can influence the distribution and measurable concentrations of serum-derived inflammatory markers. As the Qalb rises, enhanced permeability, and greater protein exchange between the blood and CSF compartments, certain markers may translocate into or become more actively metabolized within the CNS, resulting in a relative decrease in serum levels [45].

From a prognostic perspective, the contrasting patterns observed in biomarkers emphasize the complexity of utilizing a single biomarker to predict the course of MS. Thus, rather than relying on a singular measurement, integrating serum and CSF chemokine levels may provide a more nuanced prognostic indicator. As shifts in these biomarkers may indicate distinct stages of disease activity and progression, such an integrated approach could be helpful for personalized treatment decisions in clinical practice.

Our study has several limitations. The small number of cases in the non-MS control groups and the absence of a healthy control group were the main limitations. Ethical considerations restrict lumbar punctures to cases where they are deemed medically necessary, preventing the inclusion of healthy controls. Additionally, ethical concerns limited CSF analysis to a single time point, meaning we could not perform repeated assessments.

## 5. Conclusions

In conclusion, our findings align with the literature in suggesting that chemokines are not specific to MS, although they may still serve as sensitive biomarkers. CSF CXCL13 levels were associated with the presence of new lesions on follow-up MRI scans, reinforcing its potential role as a biomarker for active disease. Also, monitoring CCL20 alongside CXCL13 may provide stronger insight. Follow-up serum CCL11 levels generally increased with an increase in EDSS, which may be a promising biomarker for predicting the risk of secondary progression.

## Figures and Tables

**Table 1:** Demographic and clinical data of MS, IIH, and OND groups.

	MS (*n* = 42)	IIH (*n* = 12)	OND (*n* = 10)	*p*
Age	31.6 ± 8.7	35.1 ± 11.9	36.3 ± 9.3	0.254 *
	17–55	18–56	21–49	
Sex (%)				
Female	29 (66%)	11 (92%)	7 (70%)	0.284 **
Male	13 (33%)	1 (8%)	3 (30%)	
Age at disease onset	29.7 ± 7.5 (16.0–46.0)			
Median disease duration in months	12.0 (0.5–204.0)			
Disease duration (%)				
<2 years ≥2 years	30 (71%) 12 (29%)			
EDSS (%)				
	1 (0–2)			
≤1 >1	31 (74%)			
	11 (26%)			

* One-Way ANOVA; ** Pearson chi-square test; Mean ± Standard Deviation; Median (minimum–maximum).

**Table 2:** CSF Characteristics of MS, IIH, and OND groups.

	MS (*n* = 42)	IIH (*n* = 12)	OND (*n* = 10)	*p*
Cell count ≤10/mm^3^ >10/mm^3^				0.250 *
	38 (88%)	12	10	
	5 (12%)	0	0	
CSF Protein (mg/dL)	37.0 ± 13.7 36.5 ^a^ (15.0–65.0)	21.7 ± 5.0 22.0 ^b^ (14.0–30.0)	31.6 ± 11.3 27.0 ^ab^ (21.0–50.0)	0.001 **
IgG index	0.77 ± 0.42 0.68 ^a^ (0.23–2.29)	0.48 ± 0.05 0.48 ^b^ (0.39–0.55)	N/A	0.021 ***
Qalb (×10^−3^)	5.54 ± 3.0 4.83 (2.29–16.70)	N/A	N/A	

* Pearson chi-square test; ** Kruskal–Wallis Test; *** Kruskal–Wallis Test; pairwise comparisons; Mean ± Standard Deviation; Median (minimum–maximum) ^a,b^: There is no difference between the groups with the same letter, N/A: Not available.

**Table 3:** Comparisons of serum and CSF chemokines according to groups.

	Groups			Test Statistics	*p*
	MS	IIH	OND		
Serum CXCL13	45.81 ± 58.07	28.05 ± 15.06	50.77 ± 69.65	1.350	0.509 *
	27.20 (17–296)	22.60 (17–65)	20.05 (17–208)		
CSF CXCL13	36.54 ± 3.39	37.38 ± 0.58	37.78 ± 0.33	8.329	0.016 *
	37.24 (16.54–38.07) ^b^	37.55 (36.36–37.99) ^ab^	37.81 (37.38–38.17) ^a^		
Serum CCL11	45.67 ± 41.91	27.09 ± 11.73	37.09 ± 27.74	0.043	0.979 *
	19.6 (15–135)	22.45 (16–50)	25.6 (15–84)		
CSF CCL11	21.11 ± 1.18	19.53 ± 2.48	20.23 ± 2.61	3.507	0.173 *
	21.47 (17.88–22.85)	20.07 (14.52–22.34)	21.24 (14.67–22.19)		
Serum CCL20	25.82 ± 1.68	26.45 ± 0.92	26.89 ± 0.99	3.555	0.169 *
	25.95 (20–28)	26.32 (25–28)	26.73 (26–28)		
CSF CCL20	28.68 ± 0.27	28.64 ± 0.34	28.9 ± 0.12	2.757	0.085 **
	28.75 (27.87–29.15)	28.64 (28.02–29.09)	28.89 (28.72–29.08)		

* Kruskal–Wallis H Test; ** One-way Analysis of Variance; Mean ± Standard Deviation; Median (minimum–maximum); ^a,b^ There is no difference between the groups with the same letter.

**Table 4:** Baseline and follow-up serum chemokine levels of MS.

	Baseline (*n* = 32)	Follow-Up (*n* = 19)	Test Statistics	*p* *
CXCL13	45.81 ± 58.07	50.53 ± 80.76	−1.409	0.159
	27.2 (17–296)	24.17 (16.54–400.78)		
CCL11	45.67 ± 41.91	56.96 ± 96.77	−0.081	0.936
	19.6 (15–135)	22.3 (14.5–436.52)		
CCL20	25.82 ± 1.68	25.62 ± 1.49	−0.242	0.809
	25.95 (20–28)	25.785 (20.15–27.63)		

* Wilcoxon Test; Mean ± Standard Deviation; Median (minimum–maximum).

**Table 5:** Investigation of the relationship between parameters within the groups.

			CXCL13	CCL11	CCL20
Baseline Serum	CXCL13	r	1		
		*p*	---		
	CCL11	r	0.669	1	
		*p*	0.000	---	
	CCL20	r	−0.025	−0.326	1
		*p*	0.879	0.040	---
CSF	CXCL13	r	1		
		*p*	---		
	CCL11	r	−0.044	1	
		*p*	0.736	---	
	CCL20	r	0.585	0.145	1
		*p*	0.000	0.261	---
Follow-Up Serum	CXCL13	r	1		
		*p*	---		
	CCL11	r	0.835	1	
		*p*	0.000	---	
	CCL20	r	−0.121	−0.264	1
		*p*	0.590	0.236	---

r = Spearman’s rho Correlation Coefficient.

**Table 6:** PATH analysis of CSF chemokine levels in the MS group (*n* = 42).

*n* = 42			β^1^	β^2^	S.Error	Test Statistic	*p*	R^2^
CXCL13	<---	Age at disease onset	0.049	0.094	0.041	1.177	0.239	0.803
CXCL13	<---	Disease duration (months)	0.001	0.012	0.007	0.155	0.877	
CXCL13	<---	CSF protein (mg/dL)	−0.033	−0.117	0.023	−1.459	0.145	
CXCL13	<---	EDSS	0.012	0.002	0.516	0.023	0.981	
		OCB negative	Reference					
CXCL13	<---	OCB positive	−0.499	−0.050	0.810	−0.616	0.538	
CXCL13	<---	IgG Index	−1.098	−0.123	0.719	−1.527	0.127	
CXCL13	<---	Qalb	0.130	0.102	0.131	0.990	0.322	
		No relapse in the first year	Reference					
CXCL13	<---	One relapse	−6.902	−0.618	0.900	−7.670	<0.001	
CXCL13	<---	Brain MRI T2 WM#	0.028	0.091	0.025	1.134	0.257	
		No new lesion on the follow-up MRI	Reference					
CXCL13	<---	New T2 hyperintense lesion on follow-up MRI	5.558	0.602	0.745	7.463	<0.001	
CCL11	<---	Age at disease onset	−0.018	−0.117	0.023	−0.794	0.427	0.124
CCL11	<---	Disease duration (months)	−0.001	−0.052	0.004	−0.352	0.724	
CCL11	<---	CSF protein (mg/dL)	−0.012	−0.135	0.013	−0.920	0.357	
CCL11	<---	EDSS	−0.317	−0.162	0.288	−1.103	0.270	
		OCB negative	Reference					
CCL11	<---	OCB positive	0.144	0.047	0.458	0.315	0.753	
CCL11	<---	IgG Index	0.051	0.019	0.407	0.126	0.899	
CCL11	<---	Qalb	0.052	0.134	0.074	0.706	0.480	
		No relapse in the first year	Reference					
CCL11	<---	One relapse	0.069	0.020	0.540	0.127	0.899	
CCL11	<---	Brain MRI T2 WM#	0.018	0.186	0.014	1.252	0.211	
		No new lesion on the follow-up MRI	Reference					
CCL11	<---	New T2 hyperintense lesion on follow-up MRI	0.241	0.086	0.448	0.539	0.590	
CCL20	<---	Age at disease onset	0.012	0.321	0.005	2.252	0.024	0.189
CCL20	<---	Disease duration (months)	0.000	0.053	0.001	0.375	0.708	
CCL20	<---	CSF protein (mg/dL)	0.001	0.033	0.003	0.230	0.818	
CCL20	<---	EDSS	0.013	0.029	0.064	0.206	0.837	
		OCB negative	Reference					
CCL20	<---	OCB positive	0.077	0.110	0.102	0.756	0.450	
CCL20	<---	IgG Index	0.003	0.005	0.091	0.034	0.973	
CCL20	<---	Qalb	0.019	0.212	0.016	1.159	0.246	
		No relapse in the first year	Reference					
CCL20	<---	One relapse	−0.055	−0.070	0.120	−0.454	0.650	
CCL20	<---	Brain MRI T2 WM#	0.003	0.130	0.003	0.903	0.366	
		No new lesion on the follow-up MRI	Reference					
CCL20	<---	New T2 hyperintense lesion on follow-up MRI	−0.030	−0.047	0.100	−0.302	0.763	

β^1^: Unstandardized path coefficient; β^2^: Standardized path coefficient. (Brain MRI T2 WM#: number of the T2 hyperintense white matter lesions on brain MRI).

**Table 7:** PATH analysis of serum baseline chemokine levels in the MS group (*n* = 32).

			β^1^	β^2^	S.Error	Test Statistic	*p*	R^2^
CXCL13	<---	Age at disease onset	1.135	0.088	0.454	2.499	0.012	0.985
CXCL13	<---	Disease duration (months)	−0.210	−0.098	0.075	−2.783	0.005	
CXCL13	<---	CSF protein (mg/dL)	1.029	0.144	0.251	4.099	<0.001	
CXCL13	<---	EDSS	22.003	0.136	5.664	3.885	<0.001	
		OCB negative	Reference					
CXCL13	<---	OCB positive	−36.826	−0.145	8.882	−4.146	<0.001	
CXCL13	<---	IgG Index	61.359	0.274	7.851	7.815	<0.001	
CXCL13	<---	Qalb	−19.565	−0.715	0.956	−20.455	<0.001	
		No relapse in the first year	Reference					
CXCL13	<---	One relapse	99.212	0.307	11.332	8.755	<0.001	
CXCL13	<---	Brain MRI T2 WM#	2.097	0.268	0.275	7.638	<0.001	
		No new lesion on the follow-up MRI	Reference					
CXCL13	<---	New T2 hyperintense lesion on follow-up MRI	−103.203	−0.393	9.226	−11.186	<0.001	
CCL11	<---	Age at disease onset	1.969	0.263	0.761	2.587	0.010	0.787
CCL11	<---	Disease duration (months)	−0.067	−0.054	0.126	−0.531	0.596	
CCL11	<---	CSF protein (mg/dL)	0.658	0.159	0.421	1.564	0.118	
CCL11	<---	EDSS	20.532	0.220	9.488	2.164	0.030	
		OCB negative	Reference					
CCL11	<---	OCB positive	18.033	0.123	14.948	1.206	0.228	
CCL11	<---	IgG Index	−10.835	−0.084	13.253	−0.818	0.414	
CCL11	<---	Qalb	−11.992	−0.760	1.620	−7.404	<0.001	
		No relapse in the first year	Reference					
CCL11	<---	One relapse	−13.903	−0.075	18.983	−0.732	0.464	
CCL11	<---	Brain MRI T2 WM#	0.849	0.188	0.460	1.846	0.065	
		No new lesion on the follow-up MRI	Reference					
CCL11	<---	New T2 hyperintense lesion on follow-up MRI	−2.840	−0.019	15.621	−0.182	0.856	
CCL20	<---	Age at disease onset	−0.029	−0.091	0.030	−0.986	0.324	0.816
CCL20	<---	Disease duration (months)	−0.004	−0.083	0.005	−0.904	0.366	
CCL20	<---	CSF protein (mg/dL)	−0.060	−0.335	0.016	−3.641	<0.001	
CCL20	<---	EDSS	1.153	0.288	0.369	3.129	0.002	
		OCB negative	Reference					
CCL20	<---	OCB positive	−3.760	−0.599	0.578	−6.510	<0.001	
CCL20	<---	IgG Index	1.171	0.211	0.514	2.279	0.023	
CCL20	<---	Qalb	0.098	0.144	0.063	1.555	0.120	
		No relapse in the first year	Reference					
CCL20	<---	One relapse	−0.450	−0.056	0.738	−0.610	0.542	
CCL20	<---	Brain MRI T2 WM#	0.081	0.417	0.018	4.534	<0.001	
		No new lesion on the follow-up MRI	Reference					
CCL20	<---	New T2 hyperintense lesion on follow-up MRI	−0.445	−0.068	0.610	−0.730	0.465	

β^1^: Unstandardized path coefficient; β^2^: Standardized path coefficient. (Brain MRI T2 WM#: number of the T2 hyperintense white matter lesions on brain MRI).

**Table 8:** PATH analysis of follow-up serum chemokine levels in the MS group (*n* = 19).

			β^1^	β^2^	S.Error	Test Statistic	*p*	R^2^
CXCL13	<---	Age at disease onset	6.589	0.485	1.879	3.506	<0.001	0.619
CXCL13	<---	Disease duration (months)	−0.212	−0.094	0.311	−0.681	0.496	
CXCL13	<---	CSF protein (mg/dL)	−1.609	−0.214	1.039	−1.549	0.121	
CXCL13	<---	EDSS	60.151	0.355	23.430	2.567	0.010	
		OCB negative	Reference					
CXCL13	<---	OCB positive	−13.502	−0.051	36.475	−0.370	0.711	
CXCL13	<---	IgG Index	48.959	0.209	32.358	1.513	0.130	
CXCL13	<---	Qalb	−12.197	−0.363	5.052	−2.414	0.016	
		No relapse in the first year	Reference					
CXCL13	<---	One relapse	−29.404	−0.087	46.878	−0.627	0.530	
CXCL13	<---	Brain MRI T2 WM#	0.969	0.118	1.136	0.853	0.394	
		No new lesion on the follow-up MRI	Reference					
CXCL13	<---	New T2 hyperintense lesion on follow-up MRI	15.032	0.055	37.682	0.399	0.690	
CCL11	<---	Age at disease onset	8.393	0.500	2.143	3.916	<0.001	0.689
CCL11	<---	Disease duration (months)	−0.362	−0.130	0.355	−1.018	0.308	
CCL11	<---	CSF protein (mg/dL)	−1.296	−0.140	1.185	−1.094	0.274	
CCL11	<---	EDSS	83.137	0.397	26.722	3.111	0.002	
		OCB negative	Reference					
CCL11	<---	OCB positive	−5.620	−0.017	41.601	−0.135	0.893	
CCL11	<---	IgG Index	40.850	0.141	36.905	1.107	0.268	
CCL11	<---	Qalb	−17.315	−0.417	5.652	−3.064	0.002	
		No relapse in the first year	Reference					
CCL11	<---	One relapse	−24.106	−0.058	53.466	−0.451	0.652	
CCL11	<---	Brain MRI T2 WM#	2.145	0.211	1.296	1.655	0.098	
		No new lesion on the follow-up MRI	Reference					
CCL11	<---	New T2 hyperintense lesion on follow-up MRI	16.667	0.050	42.978	0.388	0.698	
CCL20	<---	Age at disease onset	−0.045	−0.172	0.033	−1.332	0.183	0.644
CCL20	<---	Disease duration (months)	−0.004	−0.102	0.006	−0.793	0.428	
CCL20	<---	CSF protein (mg/dL)	0.007	0.051	0.018	0.395	0.693	
CCL20	<---	EDSS	1.124	0.347	0.417	2.696	0.007	
		OCB negative	Reference					
CCL20	<---	OCB positive	−2.297	−0.456	0.649	−3.539	<0.001	
CCL20	<---	IgG Index	1.196	0.268	0.576	2.077	0.038	
CCL20	<---	Qalb	0.036	0.055	0.092	0.387	0.699	
		No relapse in the first year	Reference					
CCL20	<---	One relapse	1.062	0.164	0.834	1.274	0.203	
CCL20	<---	Brain MRI T2 WM#	0.041	0.259	0.020	2.011	0.044	
		No new lesion on the follow-up MRI	Reference					
CCL20	<---	New T2 hyperintense lesion on follow-up MRI	−1.686	−0.324	0.671	−2.514	0.012	

β^1^: Unstandardized path coefficient; β^2^: Standardized path coefficient. (Brain MRI T2 WM#: number of the T2 hyperintense white matter lesions on brain MRI).

## Data Availability

Data are contained within the article.
